# Interplant carbon and nitrogen transfers mediated by common arbuscular mycorrhizal networks: beneficial pathways for system functionality

**DOI:** 10.3389/fpls.2023.1169310

**Published:** 2023-07-12

**Authors:** Xie Luo, Yining Liu, Siyue Li, Xinhua He

**Affiliations:** ^1^ School of Environmental Ecology and Biological Engineering, Institute of Changjiang Water Environment and Ecological Security, Key Laboratory for Green Chemical Process of Ministry of Education, Hubei Key Laboratory of Novel Reactor and Green Chemical Technology, Wuhan Institute of Technology, Wuhan, China; ^2^ National Base of International Science and Technology (S&T) Collaboration on Water Environmental Monitoring and Simulation in the Three Gorges Reservoir Region and Centre of Excellence for Soil Biology, College of Resources and Environment, Southwest University, Chongqing, China; ^3^ School of Biological Sciences, University of Western Australia, Perth, WA, Australia; ^4^ Department of Land, Air and Water Resources, University of California at Davis, Davis, CA, United States

**Keywords:** ^13^C, ^15^N, carbon and nitrogen cycling, interplant nutrient exchange, plant coexistence, resource competition, resource share

## Abstract

Arbuscular mycorrhizal fungi (AMF) are ubiquitous in soil and form nutritional symbioses with ~80% of vascular plant species, which significantly impact global carbon (C) and nitrogen (N) biogeochemical cycles. Roots of plant individuals are interconnected by AMF hyphae to form common AM networks (CAMNs), which provide pathways for the transfer of C and N from one plant to another, promoting plant coexistence and biodiversity. Despite that stable isotope methodologies (^13^C, ^14^C and ^15^N tracer techniques) have demonstrated CAMNs are an important pathway for the translocation of both C and N, the functioning of CAMNs in ecosystem C and N dynamics remains equivocal. This review systematically synthesizes both laboratory and field evidence in interplant C and N transfer through CAMNs generated through stable isotope methodologies and highlights perspectives on the system functionality of CAMNs with implications for plant coexistence, species diversity and community stability. One-way transfers from donor to recipient plants of 0.02-41% C and 0.04-80% N of recipient C and N have been observed, with the reverse fluxes generally less than 15% of donor C and N. Interplant C and N transfers have practical implications for plant performance, coexistence and biodiversity in both resource-limited and resource-unlimited habitats. Resource competition among coexisting individuals of the same or different species is undoubtedly modified by such C and N transfers. Studying interplant variability in these transfers with ^13^C and ^15^N tracer application and natural abundance measurements could address the eco physiological significance of such CAMNs in sustainable agricultural and natural ecosystems.

## Highlights

• Plants interconnected by arbuscular mycorrhizal (AM) fungi form common AM networks

• ^13^C and ^15^N labeling can trace the amount of AM-mediated interplant C and N transfers

• 0.02‒41% C transfers are from a donor to a receiver, but< 10% in the reverse route

• 0.04‒80% N transfers are from a donor to a receiver, but< 15% in the reverse route

• Interplant C and N transfers should enhance plant survival under nutrient-limitations

## 1 Introduction

### 1.1 Arbuscular mycorrhiza

Arbuscular mycorrhizas (AM) are formed between arbuscular mycorrhizal fungi (AMF) and roots of ~70% of ~391,000 higher plant species (Wang and Qiu, 2006; Smith and Read, 2008; Brundrett, 2009; Brundrett, 2017; Brundrett and Tedersoo, 2018). Currently 25 genera and ~338 fungal species belonging to the sub-Phylum Glomeromycota form AMF globally (Schüßler and Walker, 2010). AMF acquire soil nutrients, such as nitrogen (N), phosphorus (P) and other mineral nutrients, and transport them to their host plant in exchange for up to 20% of photosynthetically fixed carbon (C) (Smith and Read, 2008; Roth and Paszkowski, 2017). In an arbuscular mycorrhiza, the intraradical mycelium (IRM) often penetrates root cortical cells to form arbuscules, while the extraradical mycelium (ERM) extends into soil, far beyond the root zone. The ERM forages for N, P, potassium and other soil nutrients, and translocates them to the IRM, where they are exchanged for C from the host (Smith et al., 2009). The ERM is extensive enabling plant access to nutrient resources well beyond the root depletion zone (Li et al., 1991). In addition, several findings revealed that sources of carbon for mutualistic AMF include fatty acids exported from the host plants, as well as lipids and sugars (Pfeffer et al., 1999; Keymer et al., 2017; Jiang et al., 2017).

### 1.2 Common arbuscular mycorrhizal networks

**Figure 1:**
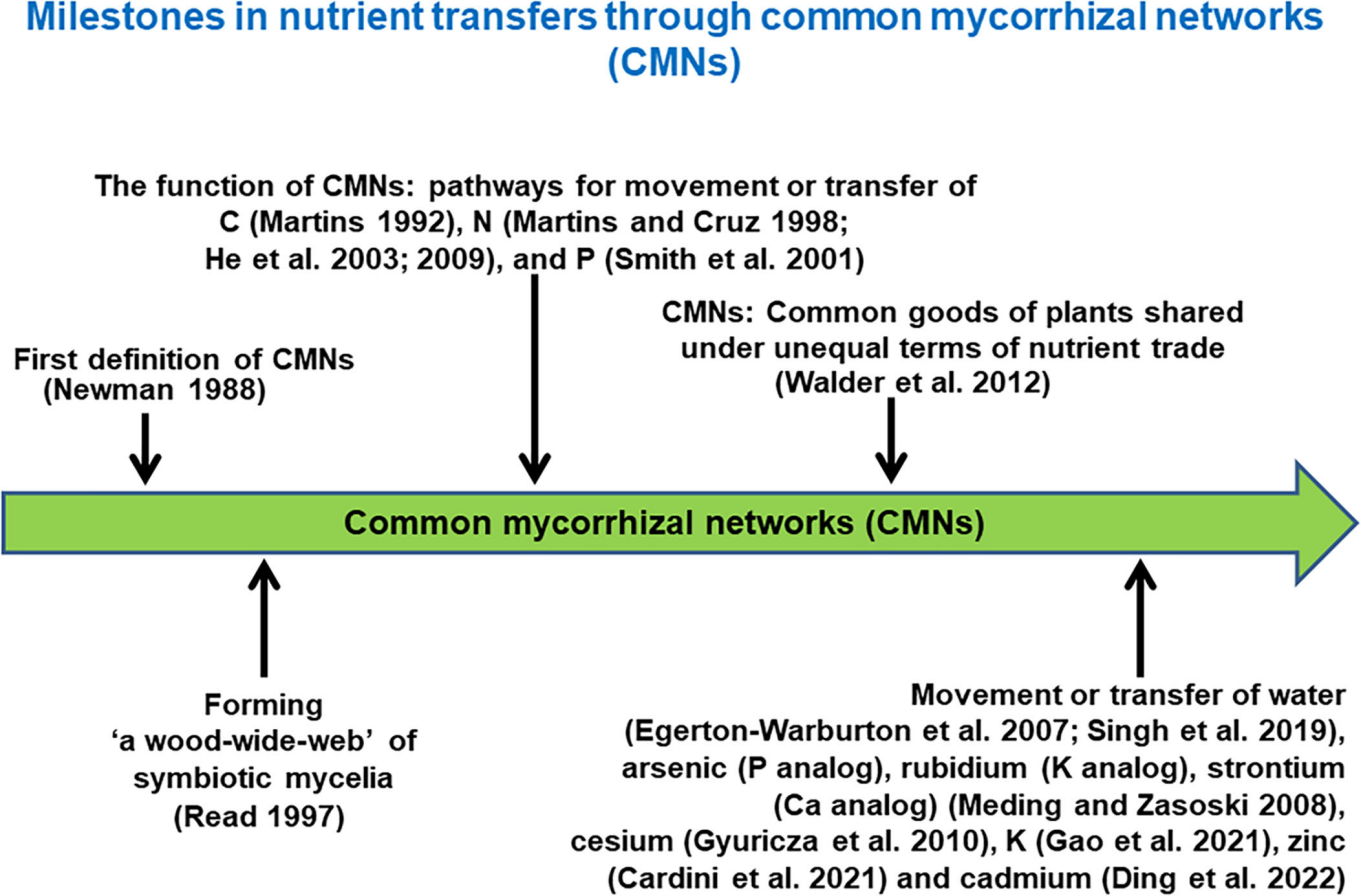
Milestones in nutrient transfers through common mycorrhizal networks (CMNs) (Note that the year of a reference pointing to the green arrow bar is not to scale).

AMF are ubiquitous components of most soil ecosystems, where they grow through soil, colonize plant roots, and can form links between plants (Newman et al., 1992; Newman et al., 1994; He et al., 2003; He et al., 2009; Molina and Horton, 2015). The plants suppling AMF with labile carbon often grow close together, primarily in multiple species communities. Because AMF exhibit little host specificity (Smith and Read, 2008), and plant roots can thus be linked by a common AM network (CAMN) (Wipf et al., 2019). Such CAMNs, being formed among individual plants of the same species or genus, or from different genera or families (Ronsheim and Anderson, 2001; Southworth et al., 2005), are usually woven into an even larger network of fungi and roots in natural communities (Smith and Read, 2008; Wipf et al., 2019). In this way, plant species within CAMNs may be joined together as a functional guild and become pathways for movement or transfer of nutrients (**Figure 1**), including C (Francis and Read, 1984; Martins, 1992; Martins, 1993; Watkins et al., 1996; Fitter et al., 1998; Mikkelsen et al., 2008; Voets et al., 2008; Walder et al., 2012), N (Hamel et al., 1991a; Hamel et al., 1991b; He et al., 2003; He et al., 2009; Frey and Schüepp, 1993; Rogers et al., 2001; Moyer-Henry et al., 2006), P (Tuffen et al., 2002; Smith and Smith, 2011; Merrild et al., 2013), arsenic (P analog, Meding and Zasoski, 2008), cadmium (Ding et al., 2022), K (Gao et al., 2021), cesium (Meding and Zasoski, 2008; Gyuricza et al., 2010), rubidium (K analogs) and strontium (Ca analog) (Meding and Zasoski, 2008, and zinc (Cardini et al., 2021). Water (Egerton-Warburton et al., 2007) and genetic material (Giovannetti et al., 2004) can also move within these networks. Movement of these materials can thus promote coexistence and biodiversity among plants (Read, 1991; Smith and Read, 2008).

Despite the considerable evidence of the functional role of CAMNs, they have not been directly visualized in natural ecosystems due to their cryptic, fragile, and microscopic nature (Newman et al., 1994; Ronsheim and Anderson, 2001; Southworth et al., 2005; Wipf et al., 2019). Plants invest photosynthetic products to feed their fungal partners, which, in return, provide mineral nutrients foraged in soil by their intricate hyphal networks (Bever et al., 2010). The Driver (AMF partners drive plant communities) and Passenger (AMF community dynamics follows changes in the host plant community) hypotheses were suggested to explain the mutual relationships of plant and AMF communities (Zobel and Öpik, 2014). Research into this complex system of plant-fungus interactions indicates that plants and fungi can choose their trading partners (Kiers et al., 2011; Walder et al., 2012).

**Figure 2:**
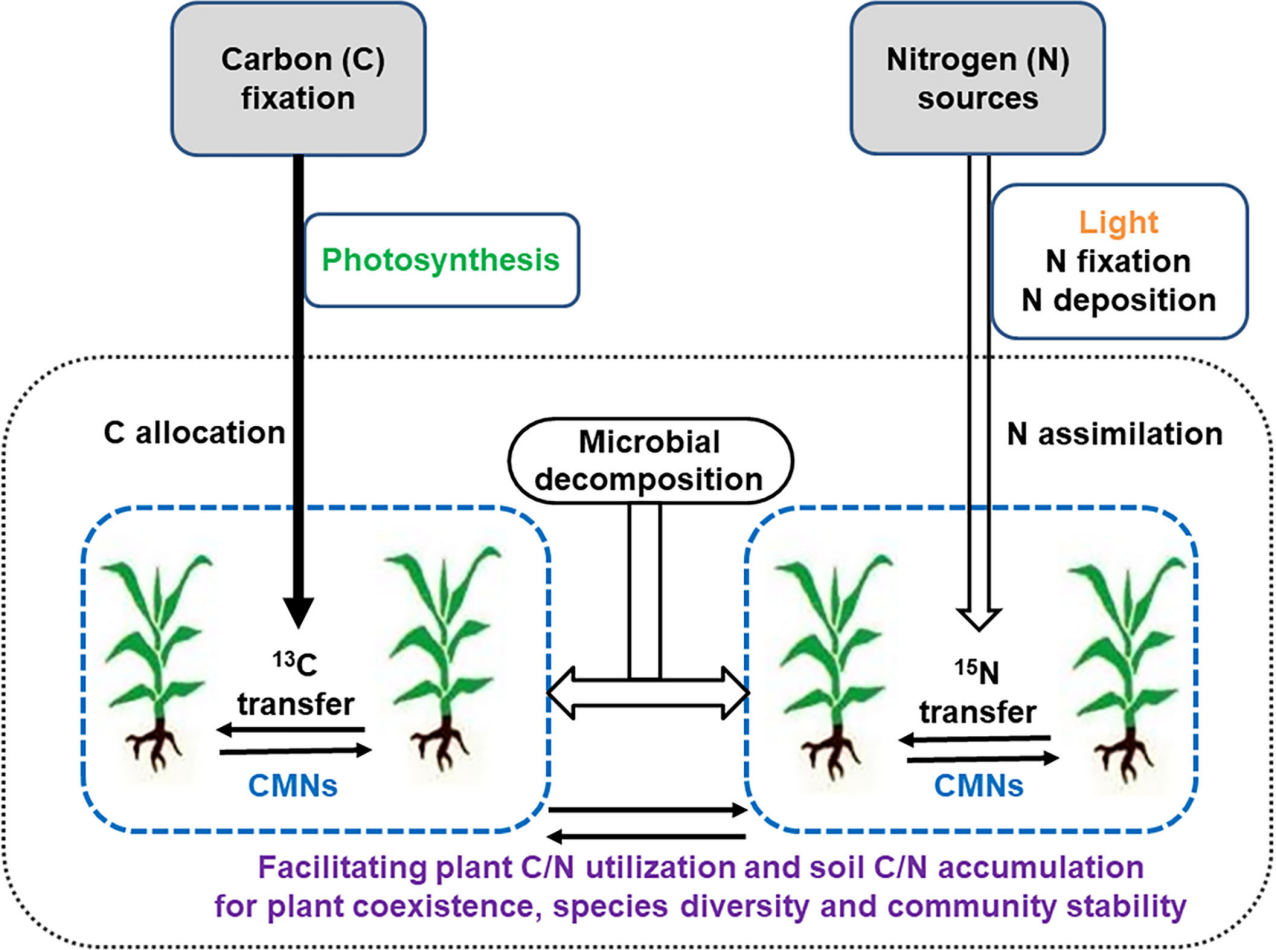
A conceptual framework of roles played by common mycorrhizal networks (CMNs) in regulating carbon (C) and nitrogen (N) flow or transfer within and between plants.

An understanding of the stoichiometry of C, N, or other nutrients mediated by CAMNs could better elucidate the potential roles of CAMNs in C and N functioning in plant-soil systems (**Figure 2**), although at present CAMNs have not been directly visualized in natural ecosystems due to their fragile and microscopic nature. Application of high-throughput genome sequences or all sorts of omics and BONCAT-FACS (bioorthogonal non-canonical amino acid tagging + fluorescence-activated cell sorting, Couradeau et al., 2019) could have an *in situ* observation of these underground cryptic microorganisms. Meanwhile, the employment of other emerging technologies, such as cryo-scanning electron microscope (Cryo-SEM), DNA stable isotope probing (DNA-SIP), quantitative multi-isotope imaging mass spectrometry (MIMS), nanoscale secondary ion mass spectrometry (NanoSIMS), single-molecule electronic device and synchrotron radiation facility, could enable the mapping the interplant flow of ^13^C and ^15^N through CAMNs. Given this demonstrated autonomy and the key role that CAMNs play in interplant nutrient transfers and biodiversity in ecosystems, it is crucial to understand how nutrient resources (e.g., C, N, P, other elements, see **Figure 1**) are shared among plants through CAMNs. And whether there may be a mechanism between CAMNs and ecosystems by which a greater biodiversity is associated with a greater productivity.

### 1.3 Application of isotopes of ^13^C and ^15^N labeling

The abundance level of stable isotopes is theoretically expressed as delta (δ) in parts per thousand or per mil (‰), which is calculated as δ^13^C or δ^15^N (‰) = [(R_Sample_/R_Standard_) –1] × 1,000, where R is the ^13^C/^12^C or ^15^N/^14^N (atom%) ratio of the sample and standard, and “Vienna”-Pee Dee Belemnite (0.0112372) or atmospheric N_2_ (0.0036765) is their respective standard material. The ^13^C and ^15^N isotopic composition (also expressed as δ^13^C and δ^15^N signatures) of plant materials can provide information on (i) inputs of photosynthetic C or uptake of fertilizer N, (ii) plant N derived from N_2_ fixation by symbiotic microorganisms, (iii) C or N cycling and (iv) the sources of N available for host plant growth (Dawson et al., 2002). For instance, the δ^13^C or δ^15^N signatures in vegetation could reflect the relative availability of C sources to fungi and N sources to plants differing in isotopic composition (Querejeta et al., 2003). Here we examine the unique and common characteristics of CMN-mediated interplant C and N transfers that are demonstrated by ^13^C and ^15^N labeling (sometimes referred to as ^13^C and ^15^N enrichment) or variations in their isotopic composition for exploring the beneficial functionality of CMNs in sustaining managed and natural systems in a changing climate (Dawson et al., 2002; He et al., 2003; Querejeta et al., 2003; Moyer-Henry et al., 2006; He et al., 2009; Jalonen et al., 2009; Kurppa et al., 2010; Walder et al., 2012; Ren et al., 2013; Meng et al., 2015; Wang et al., 2016; Řezáčová et al., 2018; Wipf et al., 2019; Muneer et al., 2020a; Alaux et al., 2021; Avital et al., 2022; Reay et al., 2022).

### 1.4 Calculation of carbon and nitrogen transfer from a donor to a receiver plant

Estimates of C or N transfer from a donor to a receiver plant are based on the assumption that an equal proportion of applied and unapplied C or N are transferred. The percentage of total C or N transferred to the receiver (% N_transfer_) is then assessed from the ratio of applied C or N in the receiver and total applied C or N in the receiver and donor. Based mostly on the calculations from Giller et al. (1991); Ikram et al. (1994); Johansen and Jensen (1996), the following equations are commonly employed by almost all relevant studies to calculate C or N transfers.


(1) $$\%\;C_{transfer}or\;N_{transfer}{=}^{13}Ccontent_{receiver}or^{15}Ncontent_{receiver}\times 100\;/\;\left(13^{\;}Ccontent_{receiver}{+}^{13}Ccontent_{donor}or^{15}Ncontent_{receiver}{+}^{15}Ncontent_{donor}\right)$$

where ^13^Ccontent_plant_ or ^15^Ncontent_plant_ = atom%^13^Cexcess_plant_ or ^15^Nexcess_plant_ ×


(2) $$total\;C_{plant}or\;N_{plant}/atom{\%}^{13}Cexcess_{labeled\;C}or\;atom{\%}^{15}Nexcess_{labeled\;N}$$

and atom%^13^Cexcess_plant_ or atom%^15^Nexcess_plant_ = atom%^13^C_plant_ or


(3) $$atom{\%}^{15}N_{plant}after\;labeling-atom{\%}^{13}C_{plant}or^{15}N_{plant}background$$

The amount of C or N (mg plant^−1^) transferred from the donor (C_transfer_ or N_transfer_) is calculated as:


$$C_{transfer}or\;N_{transfer}=\;\%\;C_{transfer}or\;\%\;N_{transfer}\times$$

(4) $$total\;C_{donor}or\;total\;N_{donor}/\;(100-\%\;C_{transfer})\;or\;(100-\%\;N_{transfer})$$

The % of C or N in the receiver derived from transfer (% CDFT or % NDFT) is calculated as:


(5) $$\%\;CDFT\;or\;\%\;NDFT\;=\;C_{transfer}or\;N_{transfer}\times 100\;/\;total\;C_{receiver}or\;total\;N_{receiver}$$

## 2 Carbon transfer between plants through common arbuscular mycorrhizal networks

### 2.1 Arbuscular mycorrhizal fungi and carbon

In the AM associations, C is the major flux from plant to fungus while P, and possibly N, are the primary fluxes from fungus to plant (Smith and Read, 2008). In general, 5–20% of plant assimilated net C was transferred between linked plants via the CAMNs (Pearson and Jakobsen, 1993). Using ^13^CO_2_ to label *Hypochaeris radicata* growing in Danish coastal grasslands and tracing that labeled C for one growing season, Lekberg et al. (2013) concluded that plants allocated C to AMF even at temperatures close to freezing and that fungal structures persisted in the roots during periods of little C-allocation. Plants could release ^13^C into rhizosphere soil through AM mycelia. These results suggest that the host plant maintained a supply of C to its AMF symbionts to ensure its own ability to obtain soil mineral nutrition from the AMF’s mycelia (Fitter et al., 1998; Lekberg et al., 2010). On the other hand, C transfer via an AM network does not allow resource sharing among linked plants (Robinson and Fitter, 1999). The mycocentric view is that fungal structures within roots are parts of extended mycelia through which fungi move C according to their own C demands, not those of their autotrophic hosts (Fitter et al., 1998).

Since the growth of AMF completely depends on supply of photosynthetically fixed C from their hosts, the C supply from the plant can be regarded as an infinitely large benefit for AMF fitness (Bago et al., 2000) (**Figure 2**). From the perspective of the plant, the amount of C provided to the fungal symbiont represents the symbiotic costs (Konvalinková and Jansa, 2016). The dynamics of C exchange between plants and fungi in AM associations is conceptualized as a biological market, in which C sources are reciprocally exchanged in both directions, with preferential allocation to the partner offering the best rate of exchange (Dawson et al., 2002; Werner et al., 2014; Konvalinková and Jansa, 2016). Previous studies have suggested that there is an asymmetry in C-for-nutrient exchange between AMF and host plants. AMF acquire C from their hosts not only as carbohydrates but also as fatty acids (Pfeffer et al., 1999; Trépanier et al., 2005; Jiang et al., 2017; Keymer et al., 2017; Luginbuehl et al., 2017). It is known that the extraradical hyphae and spores of AMF secrete a special glycoprotein, glomalin into the soil, defined as glomalin-related soil protein (GRSP) (Rillig, 2004). GRSP is released to cover the surface of soil organic matter and aggregates, and can store C in protein and carbohydrate subunits, forming a protective layer that avoids the loss of nutrients (e.g., C) in soil aggregates (Schindler et al., 2007; Wang et al., 2023).

AMF manage plant-soil interactions, suppling mineral nutrients to host plants while providing a conduit of C to soil microbial community. Field studies applying ^13^CO_2_ pulse labeling have demonstrated that AMF ERM provides a rapid and important pathway of C flux from plants to soil and the atmosphere (Johnson et al., 2002). By tracing *in situ* flows of photo-assimilated C of ^13^CO_2_-exposed wheat (*Triticum aestivum*) through AMF into root- and hyphae-associated soil microbial communities over an eight-hour period, Kaiser et al. (2015) found that intraradical AMF hyphae were significantly ^13^C-enriched compared to the root-cortex area, suggesting an efflux of photosynthate C from the plant to the mycorrhizosphere over time. In addition, they showed that ^13^C photosynthate was delivered to general bacteria and Gram-negative bacteria primarily through the AM pathway rather than directly through roots. These results suggest that AMF play a vital role in the translocation of new fixed plant C to soil microbes (Kaiser et al., 2015).

### 2.2 Interplant carbon transfer through common arbuscular mycorrhizal networks

In mixed-species communities connected through CAMNs, plant species benefit differently depending on the AMF species involved and plant coexistence can be significantly affected by these differences (Wagg et al., 2011; van der Heijden et al., 2015). Differences in the natural abundance of ^13^C between plants of the C_3_ and C_4_ photosynthetic pathways were used in several studies of AMF-linked plants to quantify C transfer. In one study, C transfer of AMF-linked *Plantago lanceolata* (C_3_) and *Cynodon dactylon* (C_4_) was quantified. This varied from 0 to 41% with median at approximately 5%for individual *C*. *dactylon* plants but was not determined for *P*. *lanceolata* individuals (Watkins et al., 1996). Walder et al. (2012) found that C_3_ flax (*Linum usitatissimum*) contributed 30% of the CAMN carbon but gained up to 90% of N from the CAMNs formed by *R*. *intraradices*, which highly facilitated its growth, while the CAMN-interconnected neighboring C_4_ sorghum (*Sorgum bicolor*) contributed 70% of CAMN carbon with little N but was barely affected growth. One possible mechanism affecting these results could be the exchange of “luxury goods” between plants and AMF symbionts (Kiers and van der Heijden, 2006). Consequently, resource trading through networks of plant-AMF assemblages could be weakly reciprocal, depending on the sink strength and exchange efficiency at the symbiotic interfaces, which should differ with different plant-fungus combinations (Helgason et al., 2007). Walder et al. (2012) demonstrated that plants transferred underground resources to each other through such a mycelial network, so that nutrients could be quickly transported between different plants.

Recent observations show that mycorrhizal fungi are important regulators of C dynamics because of slow decomposition of fungal residues (van der Heijden et al., 2015) and that C storage is increased in AM-dominated ecosystems (Averill et al., 2014; van der Heijden et al., 2015; Wurzburger et al., 2017). To quantify the involvement of AMF in the intraspecific transport of C between plants, Graves et al. (1997) fumigated a mycorrhizal *Festuca ovina* turf with ^13^C-depleted CO_2_ for one week and found that 41% of the newly fixed C that was exported belowground was subsequently transported to neighbouring *F. ovina* (**Table 1**). Although Francis and Read (1984) showed that transfer of C between plants connected by AM mycelia occurred primarily by the direct hyphal pathway, levels of C in whole receiver plants (*F. ovina*) only reached to 0.058 ± 0.023% of that in donors (*Plantago lanceolata*). In contrast, by labeling plants with ^14^CO_2_ in the field, Lerat et al. (2002) reported that a direct 0.064 ± 0.049% transfer of ^14^C in the sugar maple (*Acer saccharum*) were from *Erythronium americanum*, while ^14^C was detected in 7 of 22 *E. americanum* roots from the sugar maple, with labeling in those 7 only 0.018 ± 0.021% that of sugar maple. Both the enrichment and natural abundance of ^13^C methods show one-way transfer of C between mycorrhizal plants to be 0 to 41%, in controlled or field conditions (He et al., 2003; He et al., 2009; **Table 1**). For instance, such C transfers have been detected between *Allium cepa* plants (Hirrel and Gerdemann, 1979), *Festuca idahoensis* and *Centaurea maculosa* (Carey et al., 2004), *F. ovina* and *F. ovina* (Graves et al., 1997), *Lolium perenne* and *Plantago lanceolata* (Martins, 1992), Oryza sativa and *Citrullus lanatus* (Carey et al., 2004), and *Trifolium subterraneum* and *P. lanceolata* (Nakano-Hylander and Olsson, 2007) (**Table 1**). Most recently, by labelling ^13^CO_2_ to one of the four tree species growing in “community boxes” using natural forest soil as fungal inoculums, 6.4 to 29.0% C transfers were facilitated by shared AMF of *R. fasciculatus* and *R. irregularis*, with oak (*Quercus calliprinos*) being a better donor, while pistacia (*Pistacia lentiscus*) and cypress (*Cupressus sempervirens*) better recipients (Avital et al., 2022). They concluded that an asymmetric C exchange between co-existing plant species could contribute to forest resilience. However, the mechanism of C transfer and role of mycorrhizal hyphae in the direct transfer of C are not well established (Robinson and Fitter, 1999; Smith and Read, 2008). Therefore, more needs to be done to lay out the arguments for why and how CAMN transfer of C could contribute to the accumulation of C in ecosystems.

**Table 1:** Transfer of C from one plant to another via CAMNs (see Section 1.4 for transfer calculations).

Donor Species A	Recipient Species B	Linkage direction^*^	Inoculum involved	Substance transferred	C_transfer_ %	Reference
*Acer saccharum*	*Erythronium americanum*	A → B	Field soil	^14^C	0.06	Lerat et al., 2002
*Achillea millefolium*	*Centaurea maculosa*	A ↔ B	Field soil	—	—	Zabinski et al., 2002
*Allium cepa*	*A*. *cepa*	A → B	*Claroideoglomus etunicatum*	—	—	Hirrel and Gerdemann, 1979
*Calamagrostis epigejos*	*C. epigejos*	A ↔ B	*Funneliformis mosseae* *C*. *claroideum*	—	—	Malcova et al., 2001
*Ceratonia siliqua*	*Ce. siliqua*	A → B	*R*. *fasciculatus*, *R*. *irregularis*	^13^CO_2_	6.35	Avital et al., 2022
*Ce. siliqua*	*Cupressus sempervirens*	A → B	*R*. *fasciculatus*, *R*. *irregularis*	^13^CO_2_	4.41	Avital et al., 2022
*Ce. siliqua*	*Pistacia lentiscus*	A → B	*R*. *fasciculatus*, *R*. *irregularis*	^13^CO_2_	29.01	Avital et al., 2022
*Daucus carota*	*D. carota*	A → B	*Rhizophagus intraradices*	^13^C / ^14^C -glucose,	—	Pfeffer et al., 2004
*D*. *carota*	*D. carota*	A → B	*R*. *intraradices*, *R*. *irregularis*	^13^CO_2_	—	Lekberg et al., 2010
*Digitalis purpurea*	*Dactylis glomerata*	A ↔ B	Field soil	—	—	Newman et al., 1994
*E*. *americanum*	*A*. *saccharum*	A → B	Field soil	^14^C	—	Lerat et al., 2002
*Festuca idahoensis*	*Centaurea maculosa*	A → B	Field soil	—	—	Zabinski et al., 2002
*Festuca idahoensis*	*Centaurea maculosa*	A → B	Field soil	^12^C	15.00	Carey et al., 2004
*Festuca ovina*	*Briza media*	A → B	*Septoglomus constrictum*	^14^C	—	Grime et al., 1987
*F*. *ovina*	*Centaurea nigra*	A → B	*S*. *constrictum*	^14^C	—	Grime et al., 1987
*F*. *ovina*	*Centaurium erythraea*	A → B	*S*. *constrictum*	^14^C	—	Grime et al., 1987
*F*. *ovina*	*F*. *ovina*	A → B	AM root segments	^13^C	41.00	Graves et al., 1997
*F*. *ovina*	*F*. *ovina*	A → B	*S*. *constrictum*	^14^C	—	Grime et al., 1987
*F*. *ovina*	*Hieracium pilosella*	A → B	*S*. *constrictum*	^14^C	—	Grime et al., 1987
*F*. *ovina*	*Leontodon hispidus*	A → B	*S*. *constrictum*	^14^C	—	Grime et al., 1987
*F*. *ovina*	*Plantago lanceolata*	A → B	*S*. *constrictum*	^14^C	—	Grime et al., 1987
*F*. *ovina*	*Poa pratensis*	A → B	*S*. *constrictum*	^14^C	—	Grime et al., 1987
*F*. *ovina*	*Scabiosa columbaria*	A → B	*S*. *constrictum*	^14^C	—	Grime et al., 1987
*Flaveria bidentis*	*Setaria viridis*	A Δ B	*R*. *intraradices*	^13^C	2.54‒2.67	Chen et al., 2021
*Flaveria bidentis*	*Eclipta prostrata*	A Δ B	*R*. *intraradices*	^13^C	3.26‒3.37	Chen et al., 2021
*Juglans nigra*	*Zea mays*	A → B	Fileld soil and root	^13^C	—	van Tuinen et al., 2020
*Lolium perenne* (Full light)	*Pl*. *lanceolata* (Full light)	A → B	AM root segments	^14^C	0.09	Martins, 1992
*L. perenne* (Dark)	*Pl*. *lanceolata* (Dark)	A → B	AM root segments	^14^C	0.27	Martins, 1992
*Lolium perenne*	*L*. *perenne*	A → B	AM root segments	^14^C	—	Martins, 1993
*Lotus corniculatus*	*Lotus corniculatus*	A ↔ B	Fileld soil and roots	^14^C	1.20	Waters and Borowicz, 1994
*Medicago truncatula*	*M*. *truncatula*	A → B	*R*. *intraradices*	^13^C	—	Voets et al., 2008
*Oryza sativa*	*O*. *sativa*	A → B	*F*. *mosseae*	^14^C	—	Ren et al., 2013
*O*. *sativa*	*Citrullus lanatus*	A → B	*F*. *mosseae*	^14^C	—	Ren et al., 2013
*Plantago lanceolata*	*Cynodon dactylon*	A → B	AM root segments	δ^13^C	10.00	Watkins et al., 1996
*Pl*. *lanceolata*	*C*. *dactylon*	A → B	*F*. *mosseae*	δ^13^C	—	Fitter et al., 1998
*Pl*. *lanceolata*	*F*. *ovina* (Full light)	A → B	*C*. *caledonium* or Field root pieces	^14^C	0.02	Francis and Read, 1984
*Pl*. *lanceolata*	*F*. *ovina* (Half light)	A → B	*C*. *caledonium* or Field root pieces	^14^C	0.05	Francis and Read, 1984
*Pl*. *lanceolata*	*F*. *ovina* (Dark)	A → B	*C*. *caledonium* or Field root pieces	^14^C	0.11	Francis and Read, 1984
*Pl*. *sativum* cv. Frisson and P2	*Triticum* × *Secale*	A → B	AMF inoculum	^13^C-glucose	1.08	Hupe et al., 2021
*Quercus calliprinos*	*Cu. sempervirens*	A → B	*R*. *fasciculatus*, *R*. *irregularis*	^13^CO_2_	15.09	Avital et al., 2022
*Q. calliprinos*	*Pi. lentiscus*	A → B	*R*. *fasciculatus*, *R*. *irregularis*	^13^CO_2_	27.12	Avital et al., 2022
*Trifolium subterraneum*	*Pl. lanceolata*	A → B	*R*. *intraradices*	Organic C	—	Nakano-Hylander and Olsson, 2007

*Symbols indicate the direction of nutrients transferred: → unidirectionally, Δ bi-directionally, ↔ either direction. Updated from He et al. (2003; He et al., 2009).

## 3 Nitrogen transfer between plants through common arbuscular mycorrhizal networks

### 3.1 Arbuscular mycorrhizal fungi and nitrogen

In contrast to C, AMF were previously thought to play no roles in organic N acquisition for their host plant (Read, 1991). In ecosystems where decomposition and nitrification processes are favored, although both poorly mobile ammonium (NH_4_^+^) and highly mobile nitrate (NO_3_^−^, most available in non-waterlogging habitats, compared to NH_4_^+^) are the principal plant-available N forms, the enhancement of plant N acquisition by AMF may be small (Read, 1991). However, studies have shown that AMF can acquire N from both inorganic and organic N sources and transfer some of this N to their host plant (Johnson et al., 2002; Leigh et al., 2008; Hodge and Fitter, 2010; Hodge and Storer, 2015; Jansa et al., 2019). Since N is a key limiting nutrient in terrestrial ecosystems, if AMF enhance inorganic, organic or unspecified N uptake, this could improve host plant fitness (Hodge and Storer, 2015). In ^15^N labelling studies, inorganic (Johansen et al., 1993; Tobar et al., 1994a; Tobar et al., 1994b; Hawkins et al., 2000) and organic (Hawkins et al., 2000) N uptake by host plants is positively correlated to AMF colonization rates. In a recent ^15^N natural abundance study, 30% of total N in maize was AMF-mediated when maize grew within 5 m of N_2_-fixing *Faidherbia albida* (Dierks et al., 2021). Generally, inorganic N absorbed by the fungal ERM could be incorporated into amino acids and then transported to the fungal IRM (Johansen et al., 1996).

In most natural and productivity ecosystems most nutrient inputs to soils are as organic materials. These materials vary widely in their physical and chemical complexity, nutrient quality and quantity, and source (Read and Perez-Moreno, 2003). Single ^15^N-labelled organic materials have been used to trace the flow of N in the soil-plant system, showing that plants, or their mycorrhizal symbionts, acquired N in intact glycine (Näsholm et al., 1998). Subsequently, Leigh et al. (2008) demonstrated that *R. intraradices* could increase the N concentration of its host plant, apparently by taking up N from a decomposing patch of organic matter. AMF can obtain substantial amounts of N from decomposing organic materials, thereby enhancing plant fitness (Hodge and Fitter, 2010). However, in the absence of other microbes, there is so far no experimental evidence for any quantitative acquisition of N by AMF hyphae from organic sources (Jansa et al., 2019). On the other hand, there is partly equivocal evidence from experiments using quantum dot technology indicating that organic N could be taken up via AMF hyphae and that uptake of N in the form of certain simple amino acids was enhanced in mycorrhizal compared with non-mycorrhizal plant roots (Whiteside et al., 2012). A surprising finding, revealed through feeding ^13^C-acetate or ^15^N-Arginine to the ERM, showed that N is transported from fungus to plant as NH_4_^+^, not amino acid (Fellbaum et al., 2012). A possible mechanism to explain these observations is that arginine delivered to the fungal IRM was broken down and the NH_4_^+^ was then released to the symbiotic interface and transferred from fungus to plant ((Parniske, 2008). The external hyphae of AM fungi can directly take up ^15^NH_4_^+^ or ^15^NO_3_^–^, reduce nitrate to NH_4_^+^, and then assimilate NH_4_^+^ into the pool of free amino acids. Understanding the link between CAMN transfer of nutrient and accumulation of C and N in ecosystems is crucial to clarify potential C-for-N trades between symbionts (Hodge and Storer, 2015; Thirkell et al., 2016). Indeed, interplant nutrient exchanges could play a vital ecological role in promoting plant coexistence in ecosystems under future climatic and anthropogenic pressures with profound relevance to restoration of plant communities (**Figure 2**).

### 3.2 Interplant nitrogen transfer through common arbuscular mycorrhizal networks

Changes in plant δ^15^N values may reflect N inputs, N outputs and N isotope fractionation processes in ecosystems (Dawson et al., 2002). Use of natural ^15^N variability to investigate the role of mycorrhizae in N transfer is increasing (Dawson et al., 2002; He et al., 2003; Moyer-Henry et al., 2006; He et al., 2009). Earlier studies showed one-way AM-mediated N transfer from N_2_-fixing soybean (*Glycine max*) to non-N_2_-fixing maize (*Zea mays*) (van Kessel et al., 1985) and from N_2_-fixing *Trifolium pratense* to non-N_2_-fixing *Lolium perenne* (Haystead et al., 1988), indicating that such N transfers could be important for non-N_2_-fixing plants under N-limited conditions. Transfers of both NH_4_^+^ and NO_3_^–^ between N_2_-fixing and non-N_2_-fixing plants were mediated by CMNs (Frey and Schüepp, 1993; Johansen et al., 1996; Moyer-Henry et al., 2006). Likewise, He et al. (2006) found that N moved quickly between AM and EM mycorrhizal plant individuals in a California oak woodland, indicating a CMN-mediated nutrient distribution mechanism between plants, including the release and recapture of N from the rhizosphere soil. By foliar ^15^NO_3_ labeling to quantify N transfer between non-N_2_-fixation plants (*Eucalyptus marginata*, ectomycorrhizal (EM) species; *Melaleuca preissiana*, AM/EM species; *Verticordia nitens* AM species), Teste et al. (2015) demonstrate that plants with cluster-roots (*Banksia menziesii*, non-mycorrhizal species) or ectomycorrhizal plants were more ^15^N enriched than with AM-only plants. Nitrogen transfer was relatively high (4% of the donor plant N) among these non-N_2_-fixation plants with contrasting N-acquisition strategies. Montesinos-Navarro et al. (2016) found that CAMNs mediated N transfer between facilitated plants, suggesting that nutrient transfer through CAMNs might be a potential mechanism allowing persistent benefits for their adult facilitated plants. The potential pathways for CAMNs-mediated N transfer could be (1) direct transfer of N via connecting hyphae across the symbiotic interface, (2) increased root surface area and a reduced distance for nutrient diffusion, and (3) increased assimilation or exudation of N in the AM-colonized plants (Haystead et al., 1988; Trannin et al., 2000; He et al., 2003; He et al., 2009).

Both ^15^N enrichment studies and natural abundance measurements showed a one-way movement of 0 to 80% of receiver N from N_2_-fixing mycorrhizal to non-N_2_-fixing mycorrhizal plants, under controlled or field conditions (He et al., 2003; He et al., 2009). For instance, one-way transfer through CAMNs of ~0.09‒80% of plant N was reported in a white clover (*Trifolium repens*) ‒ ryegrass (*Lolium perenne*) system with *F. mosseae* (Haystead et al., 1988), a berseem (*Trifolium alexandrinum*) ‒ maize (*Zea mays*) or pea (*Pisum sativum*) ‒ barley (*Hordeum vulgare*) system with *R. intraradices* (Frey and Schüepp, 1993; Johansen and Jensen, 1996), a soybean (*Glycine max*) ‒ semen cassiae (*Senna obtusifolia*) and a peanut (*Arachis hypogaea*) ‒ prickly sida (*Sida spinosa*) or sicklepod (*Senna obtusifolia*) (Moyer-Henry et al., 2006), a *Cinnamomum camphora* ‒ *C*. *camphora* system with *Claroideoglomus etunicatum* (He et al., 2019), a *Vachellia seyal* ‒ *Sporobolus robustus* system with *R. irregularis* (**Table 2**). In addition, N can also be transferred from non-N_2_-fixing mycorrhizal plants to N_2_-fixing mycorrhizal plants via a CAMN, although N transfer is generally less than 10% of plant N budgets (Johansen and Jensen, 1996; Li et al., 2009). For instance, the respective N transfer was 4.2% ‒ 9.9% of plant N budgets from the donor mung bean (*Vigna radiata*) to its associated receiver rice (*Oryza sativa*) or from the rice (*O*. *sativa*) to mung bean (Li et al., 2009). AM-mediated N transfer can be from N_2_-fixing to non-N_2_-fixing plants or from non-N_2_-fixing to N_2_-fixing plants, indicating bi-directional transfer. Recently, ^15^N labeling demonstrated that CMNs increased ^15^N enrichment of *Trifolium pratense*, but did not affect its biomass production, when the holoparasite *Cuscuta australis* was absent (Yuan YG. et al., 2021). In contrast, both ^15^N enrichment and biomass production in *T*. *pratense* plants were increased by CAMNs when the holoparasite was present. These results indicated that CAMNs could preferentially distribute more N to a non-parasitized neighboring *T. pratense*, while resulting in negative feedback on the growth of the parasite *C. australis* (Yuan YG. et al., 2021).

**Table 2:** Transfer of N from one plant to another via CAMNs (see Section 1.4 for transfer calculations).

Donor Species A	Recipient Species B	Linkage direction^*^	Inoculum involved	Substance transferred	N_transfer_ %	Reference
*Andropogon gerardii* *Arachis hypogaea*	*An. gerardii* *Sida spinosa*	A → B A → B	Seven AM fungi Field soil with roots	^15^NH_4_+^15^NO_3_^−^ ^14^NH_4_^+^	27.00 30.00	Weremijewicz et al., 2016 Moyer-Henry et al., 2006
*Ar*. *hypogaea*	*Senna obtusifolia*	A → B	Field soil with roots	^14^NH_4_^+^	80.00	Moyer-Henry et al., 2006
*Bromus hordeaceus*	*Vitis vinifera*	A → B	Field soil with roots	^15^NH_4_^+^	24.80	Cheng and Baumgartner, 2004
*Cleistogene squarrosa* *Cl*. *squarrosa*	*Cl*. *squarrosa* *Leymus chinensis*	A → B A → B	Field soil with roots Root zone soil from A	^15^NH_4_^+^ ^15^NH_4_^+^	45.70‒55.30 16.00‒61.00	Muneer et al., 2020a; Muneer et al., 2020b Muneer et al., 2022
*Cinnamomum* *camphora*	*Ci*. *camphora*	A → B	*Claroideoglomus etunicatum*	^15^NH_4_^+^	0.09	He et al., 2019
*Ci*. *camphora*	*Bidens pilosa*	A → B	*C*.*etunicatum*	^15^NH_4_^+^	0.22	He et al., 2019
*Ci*. *camphora*	*Broussonetia papyrifera*	A → B	*C*. *etunicatum*	^15^NH_4_^+^	0.19	He et al., 2019
*Cupressus goveniana*	*Cu*. *goveniana*	A → B	Pygmy forest soil	^15^NH_4_^+^	0.99	Rains and Bledsoe, 2007
*Eucalyptus marginata* *Flaveria bidentis*	*Verticordia nitens* *Setaria viridis*	A ↔ B A Δ B	Nursery soil and potting media *Rhizophagus intraradices*	^15^NO_3_^−^ ^15^NH_4_^+^	2.90‒4.40 0.98‒2.14	Teste et al., 2015 Chen et al., 2021
*Flaveria bidentis*	*Eclipta prostrata*	A Δ B	*R*. *intraradices*	^15^NH_4_^+^	2.99‒4.29	Chen et al., 2021
*Leymus chinensis* *L. chinensis*	*L*. *chinensis* *Cl. squarrosa*	A → B A → B	Field soil with roots Root zone soil from A	^15^NH_4_^+^ ^15^NH_4_	21.50‒64.90 3.98‒5.98	Muneer et al., 2020a; Muneer et al.2020b Muneer et al., 2022
*Gliricidia sepium*	*Dichantium aristatum*	A → B	Field soil with roots	^15^NO_3_^−^	0.70‒2.50	Jalonen et al., 2009
*Glycine max*	*G*. *max* (non-nodulated)	A → B	Field soil with roots	^14^NH_4_^+^	48.00	Moyer-Henry et al., 2006
*G*. *max*	*Senna. obtusifolia*	A → B	Field soil with roots	^14^NH_4_^+^	80.00	Moyer-Henry et al., 2006
*G*. *max*	*Sorghum bicolor*	A → B	*F*. *mosseae*	^14^NH_4_^+^	22.50	He, 2002
*G*. *max*	*Zea mays*	A → B	3 *Glomus* species	^15^NH_4_^+^	~5.00	Hamel et al., 1991a; Hamel et al., 1991b
*G*. *max*	*Z. mays*	A → B	Field soil with roots	^15^NH_4_^+^	3.00	Eissenstat, 1990
*G*. *max*	*Z. mays*	A → B	*R*. *irregularis*	^15^NH_4_^+^	11.40	Wang et al., 2016
*G*. *max*	*Z. mays*	A → B	*R*. *fascculatus*	^15^NH_4_^+^	—	van Kessel et al., 1985
*G*. *max*	*Z. mays*	A → B	*Funneliformis mosseae*	^15^NH_4_^+^	6.08	Meng et al., 2015
*Gliricidia sepium*	*Theobroma cacao*	A → B	Field soil with roots	^15^NH_4_^+^	0.40–0.85	Kurppa et al., 2010
*Hordeum vulgare* (barley)	*Pisum sativum*	A → B	*R*. *intraradices*	^15^NH_4_^+^	4.00	Eissenstat, 1990
*Inga edulis*	*T*. *cacao*	A → B	Field soil with roots	^15^NH_4_^+^	0.55–0.88	Kurppa et al., 2010
*Kummerowa striata*	*Solidago canadensis*	A → B	*Acaulospora scrobiculata* *Gigaspora margarita* *F*. *geosporum*	^15^NO_3_^−^	—	Awaydul et al., 2019
*Medicago polymorpha* *Melaleuca preissiana*	*Vitis vinifera* *V. nitens*	A → B A ↔ B	Field soil with roots Nursery soil and potting media	^15^NH_4_^+^ ^15^NO_3_^−^	5.50 2.90‒4.40	Cheng and Baumgartner, 2004 Teste et al., 2015
*Oryza sativa* (Rice)	*Vigna radiate* (Peanut)	A → B	*Claroideoglomus caledonium*	^15^NH_4_^+^	1.40‒4.40	Li et al., 2009
*Phaseolus vulgaris*	*Zea mays*	A → B	*F. mosseae*	^15^NH_4_^+^	0.32	Giller et al., 1991
*Pisum sativum* (pea)	*Cichorium intybus*	A → B	*R. irregularis + F. mosseae*	^15^NH_4_^15^NO_3_^−^	52.50	Ingraffia et al., 2021
	*Linum usitatissimum*	A → B			13.40	
	*Triticum durum*	A → B			34.00	
*Pi. sativum*	*Hordeum vulgare* (barley)	A Δ B	*R*. *intraradices*	^15^NH_4_+^15^NO_3_^−^	15.00	Johansen and Jensen, 1996
*Pi*. *sativum cv.* Frisson and P2	*Triticum* × *Secale*	A → B	AMF inoculum	^15^N-urea	0.67	Hupe et al., 2021
*Plantago lanceolata*	*Pl*. *lanceolata*	A ↔ B	Filed soil	^15^NH_4_^+^	0.70	Eissenstat, 1990
*Pl*. *lanceolata*	*Pl*. *lanceolata*	A ↔ B	*Glomus hoi*	^15^N organic patch	—	Hodge and Fitter, 2010
*Pl*. *lanceolata*	*Pl*. *lanceolata*	A ↔ B	*F*. *mosseae*	^15^N organic patch	—	Hodge and Fitter, 2010
*Pueraria phaseoloides*	*Hevea brasiliensis*	A → B	*C*. *clarum*	^15^NO_3_^−^	0.04‒0.20	Ikram et al., 1994
*Sorghum bicolor*	*Glycine max*	A → B	*F*. *mosseae*	^15^NH_4_^+^	28.50	He, 2002
*Sesbania virgata*	*Eucalyptus grandis*	A → B	*G*. *macrocarpum*, *G*. *etunicatum* *Entrophospora colombiana*	^15^NH_4_^+^	0.06‒0.08	Rodrigues et al., 2003
*Trifolium alexandrinum*	*Malus domestica*	A → B	*R*. *intraradices*	^15^NH_4_^+^	4.70	Frey and Schüepp, 1993
*Trifolium repens*	*Citrus sinensis* Osbeck	A → B	*R*. *intraradices*	^15^NH_4_^+^	1.40‒1.70	Fang et al., 2021
*T. repens* *T. repens*	*Lolium perenne* *l. perenne*	A ↔ B	*F*. *mosseae* *R. irregularis)*	^15^NH_4_^+^ ^15^-urea	4.20–5.00 2.00–3.00	Haystead et al., 1988 Reay et al., 2022
*Vachellia seyal*	*Sporobolus robustus*	A → B	*R*. *irregularis*	^15^NH_4_^+^	13.90	Fall et al., 2022
*Verticordia nitens*	*Melaleuca preissiana*	A ↔ B	Nursery soil and potting media	^15^NO_3_^−^	2.90–4.40	Teste et al., 2015
*Vicia faba*	*Triticum durum*	A → B	8 AMF species	^15^NH_4_^15^NO_3_	2.00‒2.70	Ingraffia et al., 2019
*V*. *faba*	*Triticum turgidum*	A → B	*R*. *irregularis*	^15^NH_4_^+^	32	Wahbi et al., 2016
*V*. *faba*	*T*. *turgidum*	A → B	*R*. *irregularis*	^15^NH_4_^+^	50	Wahbi et al., 2016
*Vigna radiate* (Peanut)	*Oryza sativa* (Rice)	A → B	*C*. *caledonium*	^15^NH_4_^+^	4.20‒9.90	Li et al., 2009
*Vigna unguiculata*	*Z*. *mays*	A → B	*C*. *etunicatum*	^15^NH_4_^+^	21.20	Martins and Cruz, 1998
*Z. mays* *Z*. *mays*	*M. sativa* *T*. *alexandrinum*	A → B A → B	Field study *R*. *intraradices*	^15^N-urea ^15^NH_4_^+^	7-10 0.10	Zhang et al., 2019 Frey and Schüepp, 1993

*Symbols indicate the direction of nutrients transferred: → unidirectionally, Δ bi-directionally, ↔ either direction. Updated from He et al. (2003; He et al., 2009).

## 4 Conclusions and future perspectives

A range of 0.02 to 41% (C) and 0.04 to 80% (N) of one-way transfer have been observed from donor to recipient plants through the determination of ^13^C and ^15^N signatures (**Tables 1**, **2**). Interplant C and N transfers can affect not only the growth and competition between donor and recipient plants but also ecosystem stability. For example, Weremijewicz et al. (2016) observed that *Andropogon gerardii* plants in intact CMNs under sunlight acquired 9% of their N, but shaded plants (~35% photosynthetically active radiation) acquired only 1% N, from their conspecific neighbors. They suggested that AM fungi in CAMNs preferentially provide N to conspecific hosts of with fixed C or presenting the strongest sinks, thus potentially expanding asymmetric underground competition. Castro-Delgado et al. (2020) showed that the mycelium could transfer diverse compounds and signals among plants that would modify plant behavior in favor of protection of the whole network. In general, stable isotope tracing has provided an effective way to study the exchange of mineral nutrients between plants through CAMNs. Although ^13^C and ^15^N labeling techniques have demonstrated that CAMNs are an important pathway for the translocation of both C and N, the functioning of CAMNs in ecosystem C and N dynamics remains equivocal. To make an explicit link between nutrient transfer in CAMNs and nutrient cycling in ecosystems new approaches are needed. For example, a combination of high-throughput genome sequence techniques with model-based assessments could further identify the extent of CAMNs in interplant C and N translocation in natural and managed ecosystems (Orwin et al., 2011; Zhou et al., 2021). The following issues about the physiological and ecological functions of AMF or CAMNs should be addressed.

1. Can ^13^C and ^15^N natural abundance, like ^13^C and ^15^N external labeling, be employed to detect C and N transfer? Study have shown that plant δ^13^C signatures could reflect the δ^13^C of the C sources of associated fungi and δ^15^N signatures could reflect the δ^15^N of N sources to plants (Querejeta et al., 2003). However, the reliability of using ^15^N natural abundance to estimate AMF-mediated N transfer has been recently questioned (Choi et al., 2020; Jach-Smith and Jackson, 2020).
2. In what form are C and N transferred through CAMNs? Amino acids, lipids, or carbohydrates for C, amino acids or ammonium for N? Does a pollen development encompass a mechanism that is shared with CAMNs symbiosis? What may the two phenomena have in common (Nouri and Reinhardt, 2015)? Can fluorescent nanoscale semiconductors or quantum dots (Whiteside et al., 2012) be combined with ^13^C and ^15^N labelling to trace the transfer of organic nutrients through CAMNs (Govindarajulu et al., 2005; Parniske, 2008)?
3. Can network theory and computer modeling (Southworth et al., 2005; Wipf et al., 2019) simulate the direction and distribution of interplant C and N transfer facilitated by CMNs and thus predict both positive and negative effects of CMNs in natural and managed systems (Alaux et al., 2021)?
4. ^15^N labeling showed that AMF could not directly decompose organic matter, but the interaction between AMF and other decomposers enhanced organic matter decomposition and hence the absorption of N by AMF (Hodge and Fitter, 2010). However, how can CAMNs regulate the process of C and N translocation and absorption between AMF mycelia and host plants? In addition, a coupled concurrent C and N movement through CAMNs has not been reported.
5. What determines the net effect of CAMN-mediated interplant nutrient transfer on plant C assimilation and N metabolism? Does the transferred C and N affect the performance or fitness of the donor, receiver, or both? What is the ecological significance of CAMN mediated nutrient transfers in natural and managed ecosystems? Whether AMF-mediated interplant C and N transferred is agronomically important to managed ecosystems, including agroforestry, forestry, croplands, and grasslands, is debated (Rillig et al., 2019; Ryan et al., 2019). How do modern agricultural practices, such as long-term organic farming, no-till, or fertigation affect the establishment and performance of CMNs and subsequent effects on fertilizer use efficiency, crop agronomic characters and productivity?
6. How the abundance and function of soil bacterial and other fungal communities could be manipulated and promoted through a CAMN-mediated interplant C and N transfers (Bonfante and Anca, 2009; Yuan MTM. et al., 2021)? Is plant C investment in AM fungal growth related to soil N acquisition within a CAMN? How is the N for C trade between mycorrhizal symbionts regulated if plants are linked through a CAMN? What determines the magnitude and direction of such C and N transfer within the same or different plant species in mono-species or mixed-species systems, particularly along their complete plant growth and development cycle? How exogenous and endogenous factors can interplay with CAMNs, and how a nutrient can impinge on AM symbiotic signaling and also on a later cellular program in host plants (Nouri et al., 2014).
7. Irrespective of photosynthetic capabilities or N_2_-fixation characteristics of plant species, what the phylogenetic and functional diversity of plant species can benefit from nutrient transfer through CAMNs? These species would be in a diverse range as C_3_, C_4_, C_3_-C_4_, CAM and parasitic plants. Are there interactions between AM and EM networks on C and N transfers since some plants do have dual AM/EM associations (Wang and Qiu, 2006; Teste et al., 2015)? How can technical problems be overcome in demonstrating unequivocally that a C or N transfer directly occurs through CMNs rather than indirectly through root exudates or soils (Zhang et al., 2019; Fall et al., 2022; Reay et al., 2022)?
8. How will drivers of global environment change including elevated CO_2_ concentration, N deposition, drought and temperature affect interplant C and N transfer through CAMNs? Each can have substantial impacts on the direction and magnitude of such C and N transfers and ultimately on resource sharing or competition (Fellbaum et al., 2014; Řezáčová et al., 2018; Mickan et al., 2021). To answer these issues, it is important to keep in mind that mycorrhizal symbiotic benefits are interactively formed between plants and fungi under specific habitats and soil properties.

## Author contributions

Conceptualization, XH. Methodology, XH and SL. Data analysis, XL and YL. Original draft, XL and XH. Review and editing, XH, XL and SL. Funding acquisition, XH and XL. All authors contributed to the article and approved the submitted version.
